# Non-Covalent Associates of siRNAs and AuNPs Enveloped with Lipid Layer and Doped with Amphiphilic Peptide for Efficient siRNA Delivery

**DOI:** 10.3390/ijms19072096

**Published:** 2018-07-19

**Authors:** Julia Poletaeva, Ilya Dovydenko, Anna Epanchintseva, Kseniya Korchagina, Dmitrii Pyshnyi, Evgeny Apartsin, Elena Ryabchikova, Inna Pyshnaya

**Affiliations:** Institute of Chemical Biology and Fundamental Medicine, Siberian Branch of Russian Academy of Sciences, Novosibirsk 630090, Russia; fabaceae@yandex.ru (J.P.); dovydenko.il@gmail.com (I.D.); annaepanch@gmail.com (A.E.); ksenkor1985@yandex.ru (K.K.); pyshnyi@niboch.nsc.ru (D.P.); eka@niboch.nsc.ru (E.A.); pyshnaya@niboch.nsc.ru (I.P.)

**Keywords:** siRNA, non-covalent binding with AuNPs, lipid enveloped AuNPs, pH-sensitive lipidoid, GFP-silencing

## Abstract

Elaboration of non-viral vehicles for delivery of therapeutic nucleic acids, in particular siRNA, into a cell is an actively growing field. Gold nanoparticles (AuNPs) occupy a noticeable place in these studies, and various nanoconstructions containing AuNPs are reported. We aimed our work to the rational design of AuNPs-based siRNA delivery vehicle with enhanced transfection efficiency. We optimized the obtaining of non-covalent siRNAs-AuNPs cores: ionic strength, temperature and reaction time were determined. Formation of cores was confirmed using gel electrophoresis. Stable associates were prepared, and then enveloped into a lipid layer composed of phosphatidylcholine, phosphatidylethanolamine and novel pH-sensitive lipidoid. The constructions were modified with [Str-(RL)_4_G-NH_2_] peptide (the resulting construction). All intermediate and resulting nanoconstructions were analyzed by dynamic light scattering (DLS) and transmission electron microscopy (TEM) to control their physico-chemical properties. To examine the biological effect of the delivery vehicle, green fluorescent protein (GFP)-expressing human embryonic kidney (HEK) Phoenix cells were incubated with the resulting construction containing anti-GFP siRNA, with the siRNA effect being studied by flow cytometry and confocal microscopy. Transfection of the cells with the resulting construction reduced the GFP fluorescence as efficiently as Lipofectamin 3000. Thus, siRNA vehicle based on non-covalently bound siRNA-AuNP core and enveloped into a lipid layer provides efficient delivery of siRNA into a cell followed by specific gene silencing.

## 1. Introduction

Elaboration of non-viral vehicles for delivery of therapeutic nucleic acids (TNA) into a cell is one of the actively growing fields of gene therapy. Gold nanoparticles (AuNPs) hold an important place in these activities, and different variants of AuNP-based nanoconstructions, including those with a lipid coating, are reported. Lipid-AuNPs nanoconstructions were used to deliver various nucleic acids into a cell: plasmids [1,2,3], antisense oligonucleotides [4] and siRNA. siRNA has been attached onto the outer surface of a construction [5]. A unique combination of components and methods to obtain the final construction was used in each of the published works. The principal component, AuNPs, was prepared in mixture with lipids [1,6], or separately [2,5]. Meanwhile, preparation of the AuNPs core determines subsequent steps including attachment of siRNA. Unfortunately, no details of siRNA-AuNPs (siRNA-Au) preparation for lipid-covered particles were published, although many reports describing covalent binding of siRNA and AuNPs are accessible [7,8].

We suppose that the use of pre-prepared AuNPs provides the ability to control the attachment of siRNA onto AuNPs surface, and, respectively, the preparation of the nanoconstruction core. We applied non-covalent attachment of siRNA onto the AuNPs surface and have shown the stability of such construction. Furthermore, we obtained parameters of siRNA adsorption and desorption, as well as studied the siRNA degradation in a cell culture media containing 10% fetal bovine serum and in bacterial cytosol [9]. Herein, we show how it is possible to optimize preparation of non-covalent siRNA-Au associates, in particular, how to increase a number of siRNA molecules on one AuNPs.

Lipid-based nanoconstructions for TNAs delivery are considered the most promising delivery vehicles due to the lipids’ ability to fuse with cell membranes and vast room for the modification of lipid layers. Additionally, lipid layer is able to protect NAs from digestion by nucleases in blood and in other biological fluids; this feature is very important for the construction of NA-delivery systems. Properties and features of various lipids destined to assemblage of delivery-nanoconstructions are given in recent comprehensive reviews [10,11,12].

In this paper, we for the first time report the enveloping of non-covalent siRNA-Au core into a lipid layer. The composition of the nanoconstruction was optimized to provide its efficient penetration into HEK Phoenix cells followed by the silencing of a model reporter gene (GFP). We believe the approach to use non-covalently assembled siRNA-Au core to be promising, since it permits siRNA to be released from the core in the functional form [9], which is crucial for gene silencing [12,13], and could optimize amount of siRNA molecules delivered into a target cell.

## 2. Results and Discussion

Four important factors define successful gene-silencing therapy at cellular level: stability of siRNA in physiological fluids, cell targeting, overcoming endososomal isolation and release of therapeutic material from a vehicle [14]. For instance, implementation of only first three conditions can lead to low efficacy of gene silencing or even total absence of the effect. In this work, we tried to create a nanoconstruction complying with all four conditions. AuNPs bearing on their surface non-covalently attached siRNA have been chosen as a core of the construction.

Recently, we obtained non-covalent associates of siRNA and AuNPs (siRNA-Au), and studied their stability and release of siRNA from AuNP surface in biological media. The surface density of siRNA was about 50 molecules/NP (12 nm) [9], and we decided to explore the possibility to improve this value by optimization of siRNA-Au preparation.

### 2.1. Optimization of Core Formation Conditions

To optimize the preparation of non-covalent core siRNA-Au, it was necessary to determine the conditions, where the colloidal solution of the core would be stable and monodisperse, and surface density of siRNA would be as high as possible. To do this, it was necessary to determine the optimal temperature, time, concentration and salt conditions of siRNA incubation with AuNPs.

Our previous work showed that, in the case of ssDNA without thiol anchor groups, maximal surface density on AuNPs depends on a length of ssDNA and could reach up to 180 molecules/AuNP when incubating for 30 min at 56 °C in the presence of a 200-fold molar excess of ssDNA relative to AuNPs. Another necessary condition was to introduce of ssDNA into the AuNPs as-prepared suspension (just after synthesis), without changing the incubation conditions [15]. In this work, we also used as-prepared suspension of AuNPs and 200-fold molar excess of DNA relative to AuNPs to prepare siRNA-Au core.

Assemblage of associates of siRNA and AuNPs differ from assembly of ssNAs with AuNPs, because the process of NA adsorption on the surface of AuNPs is energetically more profitable than the formation of NA duplex [16,17,18]. Thus, we added pre-formed siRNA to AuNPs suspension to provide siRNA attachment to AuNP surface as duplex, not individual chains. The process of siRNA-Au core preparation was carried out at 25 °C because the incubation temperature of the AuNPs and siRNA mixture should be lower than the melting temperature of the siRNA, which, according to our data, is 44 °C in 4 mM NaCl (Vector NTI, Invitrogen, Carlsbad, CA, USA).

Addition of NaCl and siRNA mixture to the colloidal suspension of AuNPs could increase the degree of their non-covalent binding, which is used in the salt-aging method of non-covalent adsorption of ssNAs onto the surface of AuNPs [19]. On the other hand, the increase of NaCl concentration has a negative effect on the stability of the colloidal solution of citrate AuNPs [20]. Given these factors, we examined the effect of incubation time, salt conditions and different concentrations of AuNPs and siRNA (Samples I, II and III) on core formation (siRNA non-covalent binding with AuNPs). In the case of Sample II, we used as-prepared suspension of citrate AuNPs; for Sample I, the concentration of AuNPs suspension was increased; and, for Sample III, the suspension was diluted. Molar ratio of siRNA/AuNPs was maintained constant and equal to 200 at 25 °C (Table 1).

**Figure 1:**
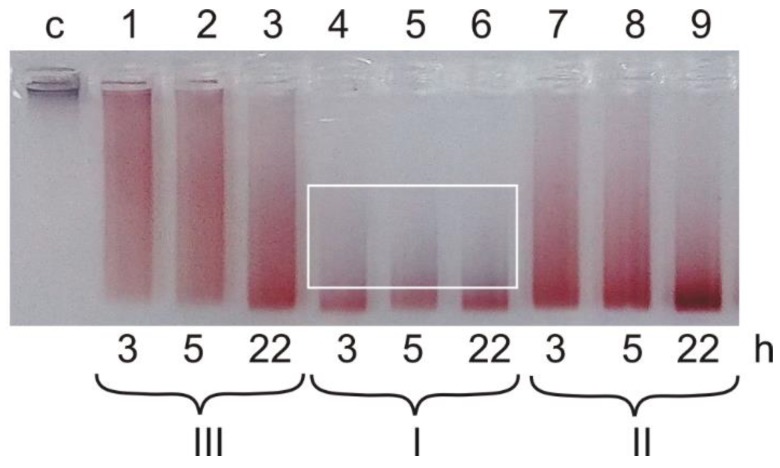
Agarose gel electrophoregram of the cores (non-covalent associates of siRNA and AuNPs), obtained by varying of component concentration and the incubation time. C, control (citrate AuNPs, not having electrophoretic mobility); I, II and III, samples of cores at different concentrations of initial components (Table 1). Incubation time is shown at the image bottom. Agglomeration area is shown by white rectangle.

The process of the core formation was analyzed by agarose gel electrophoresis, which demonstrates both the effectiveness of siRNA binding to AuNPs and the homogenicity of the colloidal suspension (Figure 1).

As we expected, the efficiency of RNA binding is directly subjected to kinetic regularities for all used concentration conditions (Figure 1 and Table 1). The increase of initial components concentration (Figure 1, Lines 4–6) leads to the rapid and efficient formation of cores. The electrophoregram shows that Sample I with an increase in incubation time to 22 h acquire a blue shade (Figure 1, Line 6) and reduced electrophoretic mobility, which indicates a possible agglomeration of nanospecies of the sample. Transmission electron microscopic (TEM) analysis revealed the presence of agglomerates.

Electrophoresis of Sample III (Figure 1, Lines 1–3) shows different mobility (blurred red lines) of negatively charged nanospecies with different degree of surface occupancy, i.e., carrying different number of bound siRNA molecules. It can be seen that, at low concentrations of siRNA and AuNPs, complete maximum mobility is not achieved even after prolonged incubation (Figure 1, line 3).

The study of Sample II (Figure 1, Lines 7–9), in which suspension of citrate-NPs was used as-prepared, revealed that it differs both from the diluted Sample III and from the concentrated Sample I. In the case of Samples II and III, the amount of nanoparticles with maximum electrophoretic mobility increases after 22 h of incubation (compare Lines 9 and 3); furthermore, there was no aggregates (blue color) in the line at the same time of incubation (compare Lines 9 and 6).

**Figure 2:**
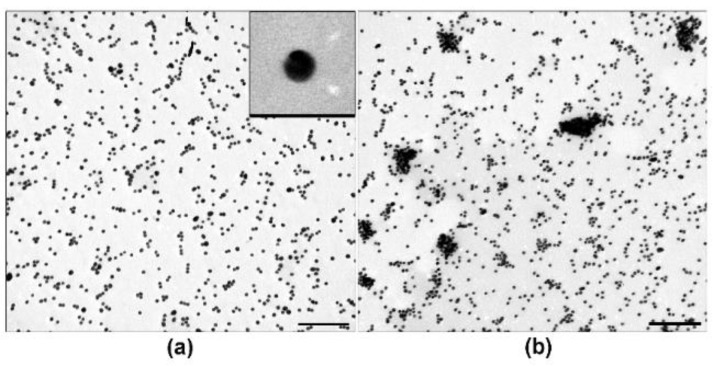
Transmission electron microscopy of cores (siRNA-Au), 22 h of incubation: Highly dispersed suspension of Sample II (**a**); and core agglomeration in Sample I (**b**). Insert shows spherical core at high magnification, length of bottom side corresponds to 40 nm. Length of scale bars corresponds to 200 nm.

We examined core Samples I, II and III under different incubation conditions by TEM. Figure 2 shows representative images of highly dispersed suspension (Figure 2a) and agglomerates of cores (Figure 2b). Agglomerates were 100–500 nm in size, and their morphology did not differ in different samples.

**Figure 3:**
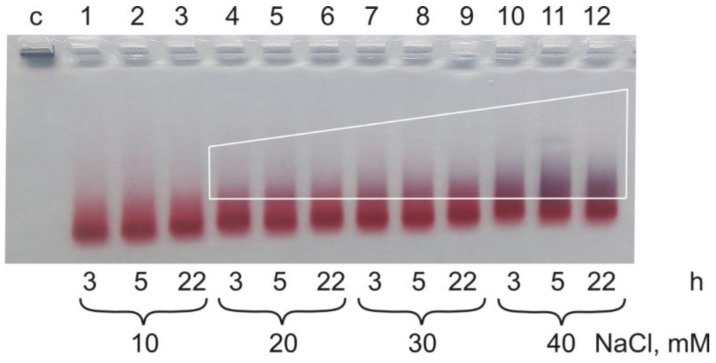
Agarose gel electrophoregram of the cores (Sample II) in the presence of salt in concentration from 10 mM to 40 mM. Agglomeration area is shown by white trapezoid. Incubation time is shown at the image bottom.

TEM showed changes in the agglomerate content in Samples I, II and III prepared in the presence of 10–40 mM salt, which corresponds to the electrophoresis data. There was no agglomeration of cores in Sample II for up to 22 h incubation after addition of 10 mM of NaCl (Figure 3, line 3). The increase of the salt concentration in Sample II from 20 to 40 mM led to the gradual core agglomeration, noticeable after 3 h of incubation detectable by the increasing of blue material in the lines (Figure 3, Lines 4–12).

**Figure 4:**
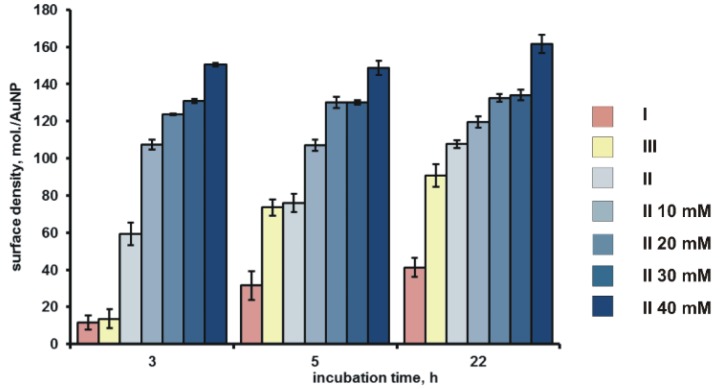
Surface density of siRNA in the composition of a core in Samples I–III. Right column shows type of a sample and NaCl concentration (10–40 mM).

Surface density of siRNA rose in all samples with the increase of incubation time from 11.8 ± 3.7 siRNA molecules/AuNP (Sample I, 3 h of incubation) to 107.7 ± 6.0 molecules/AuNP (Sample II, 22 h of incubation) (Figure 4).

It should be noted that, along with the growth of core agglomeration and with the increase of the salt concentration, the surface density of siRNA-Au increased up to 161.6 ± 4.9 molecules/AuNP (Figure 3). This value exceeds the previously obtained value of ~50 siRNA molecules/AuNP for the same non-covalent associates [9]. Thus, the optimization of the core preparation can improve the siRNA loading to the AuNPs. The surface density of siRNA duplexes on AuNP, which reaches more than 100 duplexes per one AuNP under optimal conditions of core preparation, is comparable [21] and in some cases exceeds [22] those for the constructions bearing siRNA covalently bound to AuNPs. It is difficult to discuss the data obtained, since the detailed conditions of direct binding of siRNA without thiol group to the surface of citrate covered AuNPs were not found in the literature.

Our data suggest the importance of each parameter for core assemblage (non-covalent siRNA-Au associates). There is the interplay between the surface density of the siRNA and the total colloidal stability of the system. We have chosen “medium” conditions (10 mM NaCl and 22 h) of AuNPs incubation with siRNA, to obtain the core of multilayer nanoconstruction to be used herein.

### 2.2. Assemblage of Multilayer Nanoconstruction

Free native siRNAs are rapidly digested by nucleases in the biological environment. RNAs non-covalently associated with AuNP surface also undergo degradation by nuclease-containing media [9] that calls for means of their protection. The core (siRNA-Au) could be shielded by a lipid membrane. Due to the large negative ζ-potential (−50 to −30 mV) of the core, the optimal way to envelope it into lipids is the electrostatic interaction of siRNA-Au particles with a thin lipid film bearing cationic molecules such as cationic lipids, polymers or peptides. However, the presence of molecules bearing constant positive charge may cause problems with release of siRNA from enveloped particles in endosomes. The use of pH-sensitive molecules able to change their charge depending on the ambient acidity can solve the problem of siRNA release. We have synthesized a novel pH-sensitive lipidoid to adjust the properties of lipid-enveloped nanoconstructions. Lipidoids are lipid-like molecules of various structure, which have been recently used to improve compositions of lipid-containing vehicles for the TNA delivery into a cell [10,11,23].

#### 2.2.1. The Synthesis of the pH-Sensitive Lipidoid

**Figure 5:**
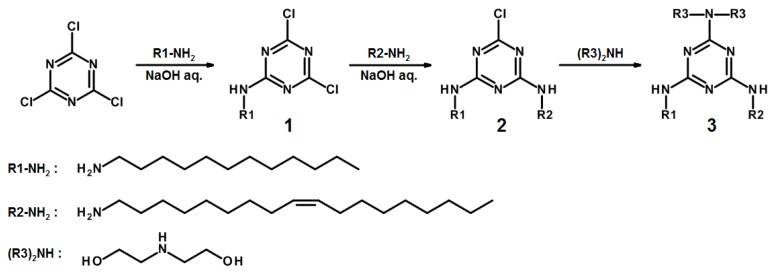
Synthesis of the pH-sensitive lipidoid (**3**).

Melamine analogs have been chosen as pH-sensitive moieties since its pK_a_ is ~5, hence we can expect its analogs to become charged at pH 4–5 (endosomes) and neutral at pH~7. Furthermore, analogs of melamine can be easily synthesized [24]. Cyanuric chloride was taken as a starting molecule for the synthesis. It was stepwise modified to obtain the desired lipidoid **3** (Figure 5).

At the first two steps of the synthesis, dodecylamine and oleylamine hydrophobic residues crucial for the integration of the lipidoid (**3**) into a lipid bilayer were introduced (Figure 5). This combination of lipophilic fragments, unlike the combination of two dodecylamine residues, provides high solubility of lipidoid in organic solvents. Furthermore, lipidoid possessing dodecylamine and oleylamine residues does not disturb a lipid bilayer as strongly as that containing two oleylamine residues [25,26]. At the final stage of the synthesis, diethanolamine was grafted to afford the desired lipidoid (**3**). Considering **3** is an amphiphile, the diethanolamine fragment serves as a hydrophilic head. The presence of two hydroxy groups in **3** is aimed increase the volume of the head thus preventing formation of micellar structures. Thus, the lipidoid (**3**) represents a pH-sensitive compound and can be added into lipid bilayer without alteration of its structure.

#### 2.2.2. Preparation of Enveloped Cores

There are several obstacles to envelop siRNA-Au into a lipid layer including aggregation of particles depending on the ionic strength, acidity of the solution, presence of organic solvents, and possibility of siRNA desorption [20,27,28]. These circumstances reduce the variety of methods available for preparation of enveloped particles. Lipid composition of nanoparticles carrying TNAs greatly varies in reported protocols [5,23,29,30]. We chose natural phospholipids, namely egg phosphatidylcholine (PC) and 1,2-dioleoyl-*sn*-glycero-3-phosphoethanolamine (DOPE) for this purpose.

**Figure 6:**
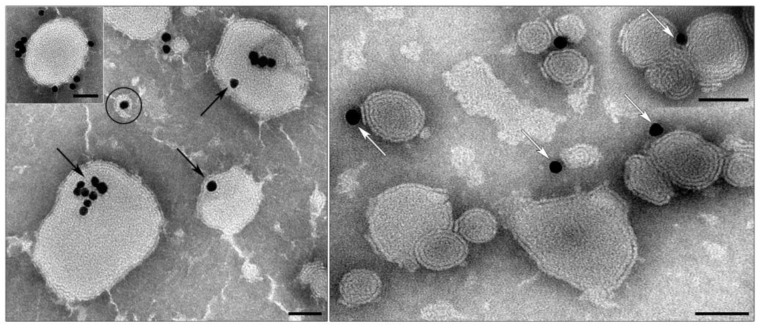
Suspension of siRNA-Au mixed with a suspension of liposomes (PC-lipidoid **3**, 9:1) in citrate buffer with pH 4.5. Black arrows show AuNPs inside liposomes; a particle of desirable morphology is outlined; white arrows show close interaction of AuNPs with liposome membranes; framed insert shows lipid particle with AuNPs on the surface. Lipid particles without AuNPs are also present. TEM, negative staining with PTA. Length of scale bars corresponds to 50 nm.

Initially, we tested a simple protocol of preparation of enveloped particles by dilution of alcohol solution of lipid mixture (PC/lipidoid **3**, in different ratios) by aqueous solution of siRNA-Au, however, it led to precipitation of siRNA-Au-LM (core-lipid mixture). The enveloping of core nanoparticles was further optimized to keep the polydispersion index as small as possible. To do this, a diluted suspension of siRNA-Au was mixed with a suspension of pre-prepared liposomes (PC-lipidoid **3**, 9:1, LM1) in the citrate buffer at pH 4.5. The idea of this approach was to assemble small lipid nanoparticles (hydrodynamic diameter 40–45 nm) containing cationic lipidoid around siRNA-Au nanoparticles having strongly negative surface charge (ζ-potential ~ −30 mV) followed by the lipid fusion to form lipid shell on siRNA-Au surface. Indeed, we observed assembling of lipid-enveloped siRNA-Au by TEM; however, this approach was not effective because of the small number of enveloped particles formed (Figure 6). In the main fraction in the suspension, there were large lipid particles bearing siRNA-Au at the surface (Figure 6, inset) and groups of lipid particles surrounding AuNPs. Many AuNPs showed close contact with membrane of lipid particles.

Thus, we have shown that lipid nanoparticles containing lipidoid **3** can interact with siRNA-Au core. We optimized the composition of lipid mixture to contain PC and lipidoid **3** in 9:1 ratio. The use of this lipid mixture (LM1) permits obtaining liposomes electrostatically interacting with siRNA-Au.

To increase the number of enveloped particles, ultrasound treatment (UT) was used. However, excessive UT leads to the desorption of siRNA from AuNPs surface, thus, first, we estimated the siRNA loss during UT, which comprised 37.0 ± 1.1% of initial siRNA surface density for 20 min at 90 W UT.

**Figure 7:**
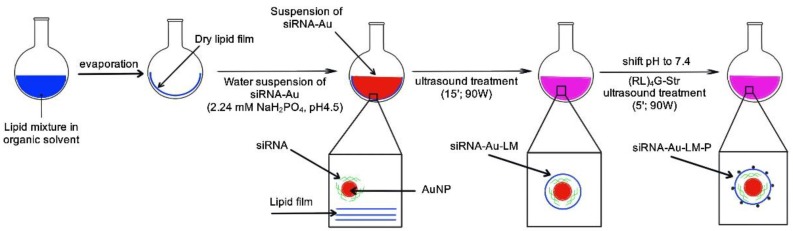
Scheme of sequential steps of nanoconstruction (siRNA-Au-LM-P) preparation.

We also found an optimal buffer for the obtaining of enveloped particles; the best results were achieved using phosphate buffer. Phosphate buffer allows working at different concentrations from 0.01 to 0.1 M and in a wide range of pH: from 4.5 to 7.5 without aggregation of siRNA-Au. Figure 7 shows a scheme of the nanoconstruction assemblage.

To obtain lipid-enveloped siRNA-Au, we prepared a thin lipid film in a flask by evaporation of LM1 solution. Then, a suspension of siRNA-Au in 2.4 mM solution of NaH_2_PO_4_ (pH 4.5) was added to thin lipid film (LM1), and the mixture was sonicated for 15 min. The resulting suspension was examined by dynamic light scattering (DLS) and TEM.

The comparative analysis of hydrodynamic diameter and ζ-potential of obtained nanoconstruction (siRNA-Au-LM1) and siRNA-Au (cores) has shown the increase of particle size from 44.9 ± 4.5 nm to 182.2 ± 20.6 nm, and shift of ζ-potential values from −30 ± 10.5 to 25.3 ± 5.1 mV. The sonicated mixture had a lower polydispersion index (PDI = 0.22), however, the preparation still contained particles with diameter of 43.8 ± 1.6 nm. This may indicate the presence of uncoated particles. The shift of pH from 4.5 to 7.4 by addition of aliquots of Na_2_HPO_4_ led to change of size from 182.2 ± 20.6 to 169.9 ± 23.7 nm (PDI = 0.24), and to decrease of ζ-potential from 25.3 ± 5.1 to −4.9 ± 4.5 mV, demonstrating that lipidoid **3** containing nancoonstruction is uncharged at physiological pH.

**Figure 8:**
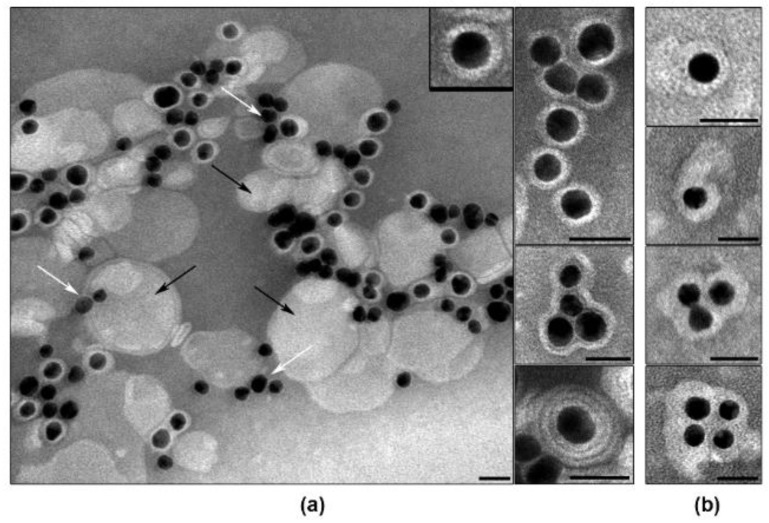
Suspension of nanospecies obtained after enveloping of siRNA-AuNPs (cores) with LM1. Black arrows show “empty” lipid particles without core, white while arrows show “naked” cores. Insert: A particle of siRNA-Au-LM1 at high magnification. Contrasting with: (**a**) PTA (pH 3.0); and (**b**) uranyl acetate. Length of scale bars correspond to 25 nm.

Examination of siRNA-Au-LM1 suspension by TEM revealed a set of nanospecies, including electron dense cores enveloped with lipid layer, “naked” cores and “empty” lipid particles (Figure 8). Individual particles containing electron dense core and lipid layer (2.5–3.5 nm) represented the main fraction in the suspension. Fine structure of a lipid layer depends on the method of contrasting: it looks blurry when contrasted with uranyl acetate (Figure 8b), whereas contrasting with PTA (pH 3.0) revealed clearly visible transverse striations and dentate outer line evidencing loosening of the structure of the lipid layer (Figure 8a). The suspension also contained particles composed of several cores and enveloped with common layer as well as individual cores covered with multilayered envelope (Figure 8). Multilayer structure was clearly seen after contrasting with PTA and was fuzzy after contrasting with UA. Thus, the visualization of the suspension obtained after UT of lipid film and siRNA-Au has confirmed the formation of individual particles composed of core and lipid envelope, and these particles made up the bulk of the preparation. 

To improve physico-chemical properties of the obtained nanoconstructions and to increase the cellular uptake of coated particles, we doped their surface with stearyl-conjugated amphiphilic peptide [Str-(RL)_4_G-NH_2_ (3 mol.%)] (P’). Comparison of DLS characteristics of two suspensions showed negligible decrease of size from 169.9 ± 23.7 nm to 156.5 ± 6.1 nm (PDI = 0.24) related to the additional UT. Doping of P’ to siRNA-Au-LM1, shifted ζ-potential value from −4.9 ± 4.5 to −11.6 ± 7.1 mV for and siRNA-Au-LM1-P’. The decrease of ζ-potential can be explained by strong interaction of guanidine groups of arginine with HPO_4_^2−^ ions in the buffer [31]. TEM examination of the nanoconstruction suspension after the doping with P’ revealed the same composition of sample as in siRNA-Au-LM1 (Figure 8). No visible changes of particle structure were detected using different contrasting agents. Thus, LM1 demonstrated good ability to envelope siRNA-Au particles due to the presence of phosphatidylcholine in the lamellar phase that permits LM1 to form envelopes around siRNA-Au particles [32]. However, the change of lipid mixture composition can dramatically affect the enveloping process. To prove that the developed approach (Figure 7) can be used for enveloping of siRNA-Au particles with any lipid of choice, we added DOPE to the original lipid mixture and obtained lipid mixture 2 (LM2). The addition of DOPE to PC in equal quantity converts lipid phase from lamellar to inverted hexagonal [33]. The interaction of siRNA-Au particles with thin lipid film consisting of LM2 led to formation of particles with hydrodynamic diameter of 158.0 ± 4.4 nm, 15.2 ± 3.7 mV ζ-potential and low value of polydispersion index (PDI = 0.195). The change of pH from 4.5 to 7.4, similar to LM1, led to small decrease in particle size from 158.0 ± 4.4 nm to 143.7 ± 4.9 nm, and the ζ-potential decreased from 15.2 ± 3.7 mV to −19.9 ± 6.0 mV. Constructions siRNA-Au-LM1 and siRNA-Au-LM2 had similar size parameters, which indicate that the presence of DOPE in LM2 does not affect the enveloping of siRNA-Au particles. The change of ζ-potential to negative at pH 7.5 can be explained by interaction of HPO_4_^2−^ ions with the ethanolamine residue of DOPE.

**Figure 9:**
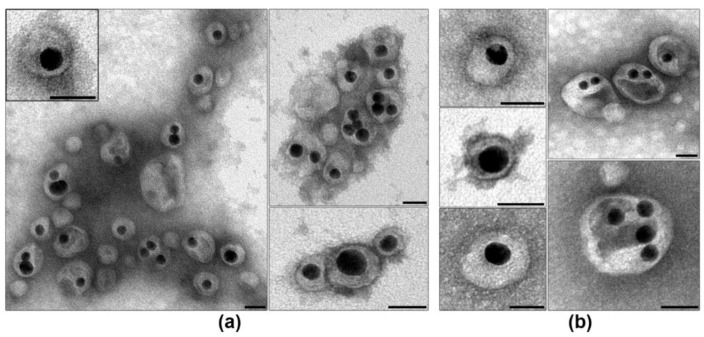
Suspension of nanospecies obtained after enveloping of siRNA-Au (cores) with LM2. Box (**a**) Note asymmetrical location of a core inside the construction, a particle at high magnification is shown in the insert. Representing images of siRNA-Au-LM2-P’ particles are shown in box (**b**). Contrasting with uranyl acetate. Length of scale bars corresponds to 25 nm.

However, TEM study of siRNA-Au-LM2 showed clear structural differences from siRNA-Au-LM1 (Figure 8a or Figure 9a). First, presence of DOPE led to loss of the stiffness and increase of the thickness of lipid layer, particles mostly have oval or irregular shape, and lipid layer often has uneven density. Electron dense core was located mostly asymmetrically in enveloped particles (Figure 9a), presumably due to the displacement during adsorption of a particle on a grid. This feature indicates the increase of the softness of lipid layer after DOPE addition and, consequently, possible increase of particle ability to fuse with lipid bilayers such s other lipid-enveloped particles and cell membranes. Presence in the suspension of particles containing several cores and empty areas (Figure 9a) could be direct consequence of increased softness of lipid layer, as well as low content of naked cores in the preparation. Thus, we have obtained soft nanoconstructions composed of dense metallic NP bearing about 150 molecules of siRNA and covered with lipid envelope representing a mixture of PC-DOPE-lipidoid **3** (4.5:4.5:1).

To complete the nanoconstruction, we doped siRNA-Au-LM2 with arginine-containing peptide P’. DLS showed tendency of decrease of particle size from 143.7 ± 4.9 nm to 127.1 ± 13.4 nm. After adding P’, the value of ζ-potential slightly changed from −19.9 ± 6.0 to −21.5 ± 6.6 mV, whereas the addition of [10 mol.% Str-(RL)_4_G-NH_2_] (P’’) decreased ζ-potential to −37.5 ± 6.7 mV. The presence of P’ did not influence particle morphology and shape in comparison with initial preparations (Figure 9a,b). Increase of peptide concentration up to 10 mol.% did not change particle morphology as well.

### 2.3. Ability of Obtained Nanoconstruction Penetrate a Cell and Influence on GFP Expression

We visualized the obtained nanoconstructions siRNA-Au-LM1-P’, siRNA-Au-LM2-P’ and siRNA-Au-LM2-P’’ as well as obtained the information on their size and charge. However, these data do not answer the question of how successfully prepared nanoconstructions deliver siRNA into the cell. To know this, we used a well-established approach to detect efficiency of siRNA delivery by specific silencing of GFP-mRNA in cell of different cultures stably expressing GFP gene [5,13,34]. First, we used confocal microscopy to compare the influence of LM composition on the efficiency of delivery of nanoconstructions into a cell.

**Figure 10:**
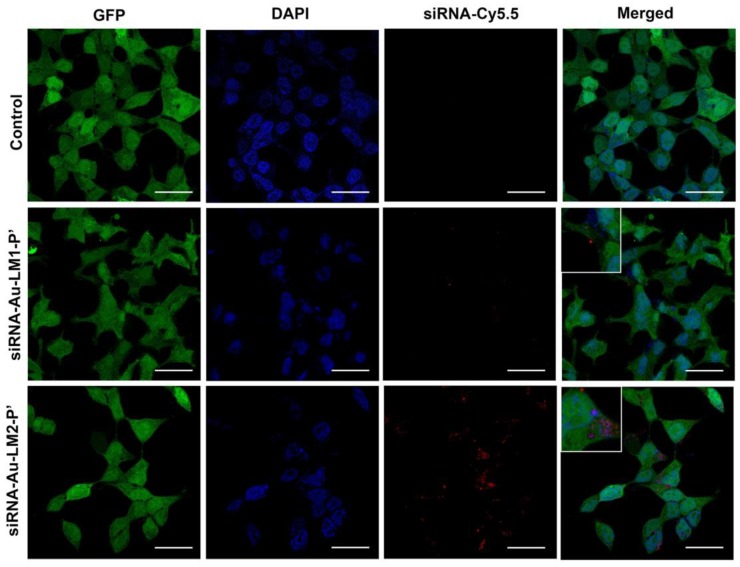
Uptake of siRNA-Au-LM1-P’ and siRNA-Au-LM2-P’ nanoconstructions by HEK Phoenix cells. siRNA was labeled with Cy5.5 and observed as red spots. Nuclei were stained blue with DAPI and green GFP was diffusely located inside whole cells. In the merged image, location of siRNA in cytoplasm was shown (red spots). Length of scale bars correspond to 20 µm.

We transfected HEK Phoenix cells stably expressing GFP with constructions bearing Cy-5.5 labeled siRNA targeted to the GFP mRNA. Transfected cells showed higher accumulation of fluorescent signal in the case of siRNA-AuNP-LM2-P’ than si-RNA-LM1-P’ (Figure 10). Since AuNPs are known to completely quench the fluorescence, the visible signal can belong only to the siRNA released from the nanoconstruction. Thus, the envelope containing DOPE provides better release of siRNA in transfected cells, and the red fluorescence is located in cytoplasm.

**Figure 11:**
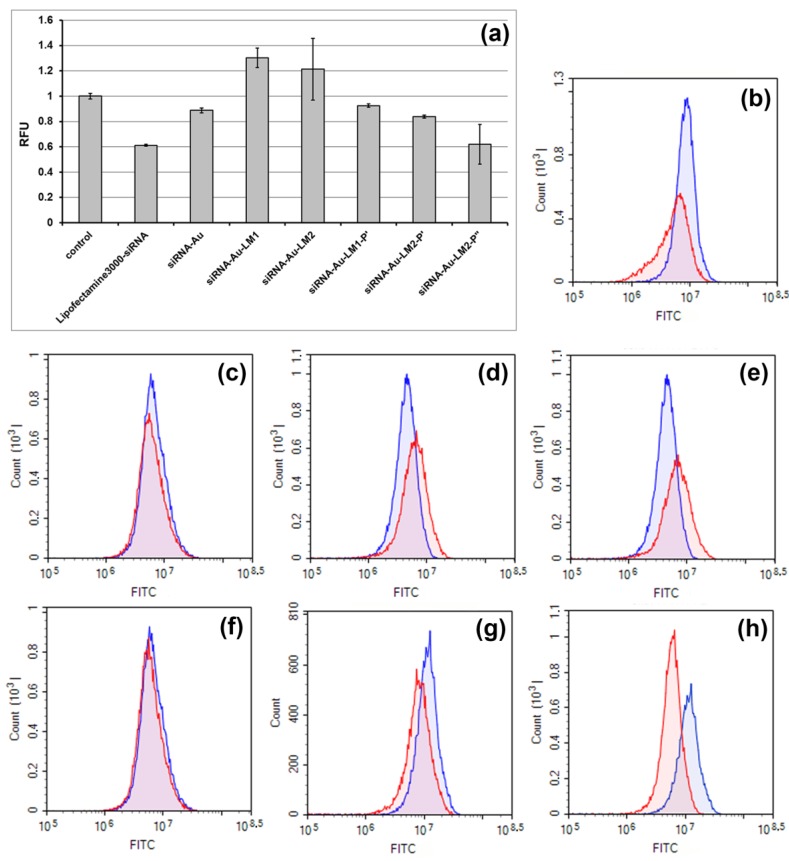
Silencing of GFP gene expression in HEK-Phoenix cells possessing stably expressed GFP by siRNA delivered with different vehicles. (**a**) The normalized mean value of the cell fluorescence (RFU = RFUexp/RFU control) after transfection with: Lipofectamine 3000 complex with siRNA (Lipofectamine3000/siRNA); cores siRNA-Au; particles siRNA-Au-LM1; siRNA-Au-LM2; siRNA-Au-LM1-P’; siRNA-Au-LM2-P’; and siRNA-Au-LM2-P’’. Example of flow cytometry analysis, cells were incubated with: Lipofectamine 3000 complex with siRNA (**b**); cores siRNA-Au (**c**); particles siRNA-Au-LM1 (**d**); siRNA-Au-LM2 (**e**); siRNA-Au-LM1-P’ (**f**); siRNA-Au-LM2-P’ (**g**); and siRNA-Au-LM2-P’’ (**h**). In blue, signal corresponding to fluorescence of non-transfected cells. More than ten thousand events were counted in each sample; mean values from three independent experiments are presented.

Then, we compared efficiency of GFP gene silencing in the cells by different nanospecies. The transfection with cores (siRNA-Au) resulted in 11.2% decrease of the GFP fluorescence (Figure 11c,f) showing slight ability of “naked” cores to deliver siRNA into cells. We used two types of lipid mixture for core enveloping, and both contained pH-sensitive lipidoid **3** necessary for trapping of siRNA-Au, containing DOPE or not. The presence of DOPE should increase efficacy of transfection due to its ability to fuse with cell membranes including endosome membrane [35]. However, constructions siRNA-Au-LM1 and siRNA-Au-LM2 did not show silencing effect (Figure 10a,d,e). Doping of these constructions with P’, which is supposed to increase transfection efficiency of enveloped particles [36], leads to the silencing observed with both siRNA-Au-LM1-P’ and siRNA-Au-LM2-P’, with the latter being twice more efficient due to the effect of DOPE (Figure 11a,f,g). The increase of peptide concentration to 10 mol.% (siRNA-Au-LM2-P’’) resulted in significant increase of silencing (GFP fluorescence was reduced up to 38%), whereas the transfection with Lipofectamine 3000 has reduced fluorescence up to 39% (Figure 11a,b,h). Substantial decrease in fluorescence caused by siRNA-Au-LM2-P’’ indicates its ability to suppress synthesis of GFP in HEK Phoenix cells.

**Figure 12:**
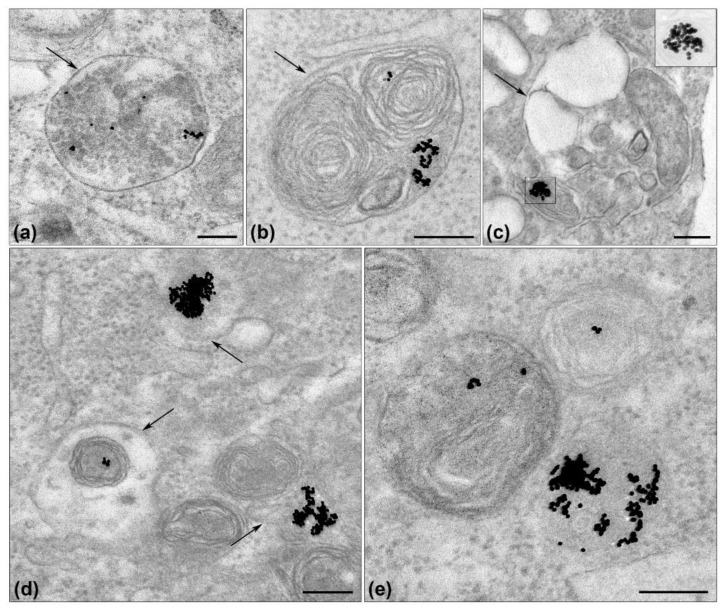
Visualization of AuNPs (serve as a marker of siRNA-Au-LM-P”) in endosome-lysosome system of HEK Phoenix cells 72 h after transfection with siRNA-Au-LM2-P’’: (**a**) late endosome; (**b**) lysosome; (**c**) autophagosome, AuNPs with low contrast are shown the insert; and (**d**,**e**) different lysosome structures. Arrows show membrane envelope of endosome-lysosome structures. Length of scale bars corresponds to 200 nm. TEM, ultrathin sections.

To know the localization of siRNA-Au-LM2P’’ in a cell, we examined ultrathin sections of same cells that were used for cytometry by TEM. The cells did not show early stages of the siRNA-Au-LM2’’ internalization due to sampling in a long time after transfection (72 h). Nevertheless, we have observed electron dense AuNPs, a marker of siRNA-Au-LM2-P’’, in late endosomes (multivesicular bodies), lysosomes and autophagosomes (Figure 12). Various lysosome-endosome structures were observed in a section of one cell (Figure 12c,d) evidencing active uptake of the particles. AuNPs were located as single particles or agglomerates only in membrane-bound endosome-lysosome structures. It is interesting that no fusion of AuNPs was detected.

Thus, the results of TEM study showed location of siRNA-Au-LM2-P’’ in endosome-lysosome structures. That means that siRNA-Au-LM2-P’’ particles penetrated the cells via endocytosis. To know the exact type of endocytosis, cell samples should be examined in a short time after transfection.

Numerous lipid-based systems for TNAs delivery have been reported showing advantages of lipid application for construction of a vehicle and TNA release in a cell [10,11,12,23]. Our study resulted with a new type of vehicle providing delivery of siRNA into a cell and specific suppression of protein synthesis. We used several approaches to meet this result, and the main feature of the nanoconstruction obtained is non-covalent binding of siRNA with AuNPs (core, non-covalent associate of siRNA and AuNP). Recently, we reported the preparation and detailed characterization of siRNA-Au, as well as proved the desorption of siRNA from AuNP surface in the form of duplex [9]. The latter is important because siRNA functioning in a cell requires RNA duplexes, and corresponding delivery represents a special issue [23,37]. In this work, we report how to optimize conditions of core preparation to increase amount of siRNA molecules non-covalently bound with one AuNP. We used layer-by-layer mode to obtain complete siRNA vehicle, when a core was enveloped with lipid mixture in contrast to some published studies when NA were attached outwardly of lipid layer [5]. Composition of lipid layer containing DOPE showed better penetration into a cell and better suppression of GFP synthesis, however, doping with amphiphilic peptide was needed to reach the level of GFP gene silencing as high as that of siRNA-Lipofectamine 3000. We suppose that the type of nanoconstruction developed can be applied in theranostic studies, due to presence of AuNPs, which are “visible” for diagnostic devices, and siRNA blocking the target protein synthesis.

## 3. Materials and Methods

### 3.1. Reagents

RNA phosphoramidites for the oligoribonucleotide synthesis were obtained from Sigma-Aldrich (Hamburg, Germany), Cy-5.5 phosphoramidite were purchased from Primetech (Minsk, Belarus). Oleylamine, dodecylamine and stearic acid were supplied from Acros Organics (Geel, Belgium). Dithiothreitol (DTT), cyanuric chloride, diethanolamine, and sodium citrate dihydrate (Na_3_C_6_H_5_O_7_) were obtained from Sigma-Aldrich (Hamburg, Germany). *N,N*-diisopropylethylamine (DIPEA) and *N*-hydroxysuccinimide (NHS) were purchased from Fluka (Seelze, Deutschland). Tetrachloroauric acid (HAuCl_4_·3H_2_O) was from Aurat (Moscow, Russia). Uranyl acetate (UA) and phosphotungstic acid (PTA) were purchased from SPI (West Chester, PA, USA). Peptide NH_2_-(RL)_4_G-C(O)NH_2_·5CF_3_COOH was obtained from Diapharm (Lyubertsy, Russia). Other chemicals and solvents were supplied by Merck (Darmstadt, Germany), Fluka (Seelze, Deutschland) and Panreac (Barcelona, Spain). Water was purified by a Simplicity 185 water system (Millipore, Burlington, MA, USA) and had a resistivity of 18.2 MΩ∙cm at 25 °C.

### 3.2. Synthesis of Lipids

#### 3.2.1. Compound **1**: 2-Dodecylamino-4,6-dichloro-1,3,5-triazine

2-Dodecylamino-4,6-dichloro-1,3,5-triazine was synthesized in accordance to [24]. Briefly, 20 mL of dodecylamine (10 mmol, 1.85 g) in chloroform were added dropwise to 20 mL of cyanuric chloride (10 mmol, 1.84 g) solution in chloroform at room temperature. Then, 8 mL aqueous solution of 5% sodium hydroxide were added to reaction mixture and reaction was kept under stirring for 30 min at room temperature. Organic phase was separated, and the solvent was removed. The resulting crude product was recrystallized from chloroform/methanol solution (1:5, *v/v*) to afford 2-dodecylamino-4,6-dichloro-1,3,5-triazine was obtained as a white solid (2.47 g, yield 74.1%).

#### 3.2.2. Compound **2**: 2-Dodecylamino-4-oleylamino-6-chloro-1,3,5-triazine

Oleylamine (4.2 mmol, 1.12 g) in chloroform (15 mL) was dropped into 15 mL solution of compound **1** (3.6 mol, 1.2 g) in chloroform followed by adding the aqueous solution of 9.5% sodium hydroxide. The reaction mixture was stirred overnight at room temperature. Organic phase was separated, and the solvent was removed. The resulting crude product was recrystallized from hexane and purified (1.84 g, yield 90.5%) 2-dodecylamino-4-oleylamino-6-chloro-1,3,5-triazine was obtained as a white solid. ^1^H and ^13^C NMR spectra were recorded on a Bruker DRX-500 spectrometer with tetramethylsilane as an internal standard. ^1^H NMR (500 MHz, CDCl_3_, δ, ppm): 0.86 (t, 6H, -*CH_3_*, oleyl, dodecyl); 1.17–1.38 (m, 40H, -CH_2_-(*CH*_2_)_9_-CH_3_, dodecyl, -CH-CH_2_-(*CH*_2_)_6_-CH_3_, -CH_2_-(*CH*_2_)_5_-CH_2_-CH-, oleyl); 1.55 (dd, 4H, -NH-CH_2_-*CH*_2_-CH_2_-, oleyl, dodecyl); 1.90–2.04 (m, 4H, -CH_2_-*CH*_2_-CH=CH-*CH*_2_-CH_2_-, oleyl); 3.38 (dd, 4H, -NH-*CH*_2_-CH_2_-, oleyl, dodecyl); 5.28–5.37 (m, 2H, -CH_2_-*CH*=*CH*-CH_2_-, oleyl). ^13^C NMR (500 MHz, CDCl_3_, δ, ppm): 14.08; 22.65; 26.84; 27.16; 29.31; 29.61; 31.88; 32.55; 40.90; 129.72; 129.93; 165.51; 167.70; 168.93.

#### 3.2.3. Lipidoid **3**: 2-[[4-Dodecylamino-6-oleylamino-1,3,5-triazine-2yl]-(2-hydroxyethyl)amino]ethanol

Diethanolamine (10 mmol, 1 mL) and compound **2** (1.8 mmol, 1 g) were dissolved in 25 mL of abs. toluene. The mixture was stirred for 6 h under reflux. Then, toluene was removed by evaporation. The residue was dissolved in 50 mL of chloroform and organic phase was washed with brine (3 × 25 mL). The organic phase was dried over Na_2_SO_4_. Following this, it was filtered and evaporated to dryness. The crude product was then purified by silica gel (30 g) column chromatography (150 mL ethyl acetate/CH_2_Cl_2_ 10/90, then 150 mL ethanol/CH_2_Cl_2_ 30/70) to afford the lipidoid **3** (0.74 g, yield 65%) as pale yellow oil. Column chromatography was performed with Silica gel 60 Å 230–400 mesh (Sigma-Aldrich, Hamburg, Germany). ^1^H NMR (500 MHz, CDCl_3_, δ, ppm): 0.86 (t, 6H, -*CH*_3_, oleyl, dodecyl); 1.18–1.35 (m, 40H, -CH_2_-(*CH*_2_)_9_-CH_3_, dodecyl, -CH-CH_2_-(*CH*_2_)_6_-CH_3_, -CH_2_-(*CH*_2_)_5_-CH_2_-CH-, oleyl); 1.50 (m, 4H, -NH-CH_2_-*CH*_2_-CH_2_-, oleyl, dodecyl); 1.89–2.06 (m, 4H, -CH_2_-*CH*_2_-CH=CH-*CH*_2_-CH_2_-, oleyl); 3.27 (m, 4H, -NH-*CH*_2_-CH_2_-, oleyl, dodecyl); 3.70 (m, 4H, -N-(CH_2_-*CH*_2_-OH)_2_); 3.82 (m, 4H, -N-(*CH*_2_-CH_2_-OH)_2_); 5.28–5.37 (m, 2H, -CH_2_-*CH*=*CH*-CH_2_-, oleyl). ^13^C NMR (500 MHz, CDCl_3_, δ, ppm): 14.08; 22.64; 26.87; 27.16; 29.32; 29.59; 31.87; 32.56; 40.75; 51.47; 62.67; 129.76; 129.88; 164.38; 165.27; 166.49.

### 3.3. Preparation of Stearyl-Modified Peptide (RL)_4_G-NH_2_

#### 3.3.1. Stearic Acid *N-*Hydroxysuccinimide Ester

Stearic acid *N-*hydroxysuccinimide ester was prepared as described in [38]. Briefly, stearic acid (3.5 mmol, 1 g), *N-*hydroxysuccinimide (3.5 mmol, 0.4 g) and dicyclohexylcarbodiimide (3.5 mmol, 0.722 g) were mixed in 20 mL ethyl acetate, and the reaction mixture was stirred overnight at room temperature. After the dicyclohexylurea was filtered off, the filtrate was concentrated, and the crude product was recrystallized from ethanol to yield (1.15 g, yield 86%) of *N-*hydroxysuccinimide ester of stearic acid.

#### 3.3.2. Synthesis of Stearyl-Modified Peptide (RL)_4_G-NH_2_ (P)

Solution of stearic acid *N-*hydroxysuccinimide ester (32 µmol, 12.2 mg) in 850 mL was added to a solution of peptide NH_2_-(RL)_4_G-C(O)NH_2_·5CF_3_COOH (3.2 µmol, 5.5 mg) in 50 µL of sterile deionized water. Reaction mixture was basified up to pH~10 by 10% NaOH (10 µL) and incubated overnight at room temperature. The reaction was monitored by TLC (isopropanol:water:NH_3_·H_2_O, 7:3:1 *v/v/v*). When all peptide was consumed, 2 mL of dichlorometane and 2 mL water was added to the reaction mixture and acidified with CF_3_COOH up to pH~2. After the extraction, water phase and interphase were gathered, 2 mL of dichlorometane were added, and the mixture was back basified with DIPEA up to pH~9. Water phase was washed twice with dichlorometane (2 mL), and organic layers were gathered and evaporated to dryness to remove volatiles and excess of organic salts. Stearate-peptide conjugate was purified by preparative silica gel chromatography in ethanol:dichloromethane (4:1) on Silica gel 60 F254 aluminum plates (Merck). Yield: 3.6 mg (79%), Rf 0.6 (isopropanol:water:NH_3_·H_2_O, 7:3:1 *v/v/v*), Rf 0.4 (ethanol:dichloromethane, 4:1). The product [Str-(RL)_4_G-NH_2_] (P) were characterized by MALDI-TOF MS analysis (theoretical mass 1418.04 Da, measured mass 1418.67 Da).

### 3.4. Synthesis of siRNA

Oligoribonucleotides were synthesized on RNA synthesis (0.4 µmol scale) was performed on the automatic ASM-800 DNA/RNA synthesizer (Biosset, Novosibirsk, Russia) by the solid-phase phosphoroamidite method using synthetic protocols [39] optimized for the instrument, with a 10 min coupling step for 2′-O-TBDMS-protected phosphoramidites (0.1 M in acetonitrile) and 5-ethylthio-H-tetrazole (0.25 M in acetonitrile) as an activating agent. Synthesis of 5′-fluorescently labeled oligoribonucleotide was carried out using Cy-5.5 phosphoramidite at the final step of the synthesis. Sequence of siRNA targeted to mRNA of GFP the sequence was taken from [40].

### 3.5. Deprotection, Purification and Annealing of siRNAs

The oligoribonucleotides ORN1 and ORN2 (Table 2) were cleaved from the support and deprotected by 40% methylamine in water at 65 °C for 15 min, and ORN3 (Table 2) deprotected by 30% aqueous ammonia at room temperature for 24 h. 2′-O-TBDMS protecting groups were removed upon treatment with a mixture of *N-*methylpyrrolidinone/TEA·3HF/TEA (150/100/75) at 65 °C for 1.5 h. Deprotected oligoribonucleotides and fluorescently labeled conjugate were isolated via preparative electrophoresis. The preparative PAGE was performed using denaturing 15% polyacrylamide gel (acrylamide:*N,N′-*methylenebisacrylamide (29:1), 8 M urea, 89 mМ Tris–borate, рН 8.3, 2 mМ Na2EDTA, 20 V/cm). After electrophoretic separation, oligonucleotide material was visualized by UV shadowing, extracted from the gel with deionized water, followed by precipitation with 2% NaClO_4_ in acetone.

The purified oligoribonucleotides were characterized by MALDI-TOF-MS. The MALDI-TOF mass-spectra were obtained with an Autoflex III (Bruker Daltonics, Bremen, Germany) mass spectrometer. Resulting siRNA duplexes were annealed at 25 µM in 0.01–1.6 M NaCl at 95 °C for 3 min.

### 3.6. Preparation of Citrate AuNPs

AuNPs were prepared using classic citrate reduction method [41]. The size and monodispersity of the obtained AuNPs were determined by transmission electron microscopy (12 ± 1 nm) and dynamic light scattering (17.3 ± 2.1 nm). A suspension of AuNPs exhibited a characteristic surface plasmon band at 520 nm. The concentration of as-prepared citrate-coated AuNPs (3 nM) was calculated from absorbance at 520 nm using extinction coefficient (8.78 ± 0.06)×10^8^ M^−1^cm^−1^.

#### Preparation of Non-Covalent Associate of siRNA and AuNPs

To obtain a core of nanoconstruction, siRNA was added to fresh AuNPs to prepare the samples.

(I) High concentration samples: 5 µL, 100 nM AuNPs, 20 µM siRNA and 10 mM NaCl.
(II) Medium concentration samples: 160 µL, 3 nM AuNPs, 0.6 µM siRNA and 10–40 mM NaCl.
(III) Low concentration samples: 1000 µL, 0.5 nM AuNPs, 0.1 µM siRNA and 0.05 mM NaCl.

All samples were stirred (1000 rpm) for 3, 5, and 22 h at 25 °C, and then gold material washed (1 mL, 3.88 mМ Na_3_C_6_H_5_O_7_). Samples were adjusted to a final volume with 3.88 mM Na_3_C_6_H_5_O_7_. The suspensions were centrifuged; sediments were analyzed separately by agarose electrophoresis. The fluorescence intensity of supernatants containing Cy5.5-labeled oligonucleotides was measured using Clariostar plate fluorimeter (BMG Labtech, Ortenberg, Germany).

### 3.7. Fluorescence Intensity Measurements and Calculation of Density of siRNA on the AuNP Surface

Density of AuNP coverage with Cy5.5-labeled siRNAs was estimated by the measurements of Cy5.5 fluorescence intensity in supernatants obtained either upon washing of AuNPs from the non-bound siRNA (Sample I) or upon desorption of siRNA from AuNPs (Sample II). The desorption was induced by the addition of dithiotreitol solution (50 mM, 100 µL) that was added to AuNP sediments followed by the gentle stirring (100 rpm) for 30 min at 56 °C.

Fluorescence measurements were done in 150 µL aliquots using Clariostar plate fluorimeter, BMG Labtech, Germany, upon excitation at 640 nm and emission detected at 700 nm. The amount of AuNP-bound siRNA was calculated from the data on the quantity of bound and non-bound oligonucleotide in Samples I and II. The siRNA surface density values were obtained taking AuNP:siRNA molar ratios into account. The measurements were run in triplicate.

### 3.8. Preparation of Lipid-Enveloped siRNA-AuNPs

To prepare lipid-enveloped siRNA-AuNPs, two types of lipid mixtures were used: LM1: egg phosphatidylcholine (PC) (Avanti, Alabaster, AL, USA) and lipidoid **3** (9:1); LM2: PC, 1,2-dioleoyl-*sn*-glycero-3-phosphoethanolamine (DOPE) (Avanti, Alabaster, AL, USA) and lipidoid **3** (4.5:4.5:1). Thin lipid films were prepared from 90 µL of 1 mM phospholipids in CHCl_3_/CH3OH (1:1) and 10 µL of 1 mM lipidoid **3** in CHCl_3_ by mixing the components in 1 mL of CHCl_3_ in a round-bottom flask; organic solvents were evaporated under reduced pressure at 25 °C. To the obtained lipid-embedded siRNA-coated AuNPs, 0.875 mL of deionized water, 22.6 µL of 1 M NaH_2_PO_4_ and 25 µL of siRNA-AuNPs (2.5 pmol of Au, 25 pmol of siRNA) were added to a lipid film, followed by ultrasound treatment (90 W) for 15 min at 20 °C. Then, to the suspensions of both nanoconstructions, 77.4 µL of 1 M Na_2_HPO_4_ were added to keep the pH at 7.4. Finally, during ultrasound treatment for 5 min, siRNA-AuNP-LM1 and siRNA-AuNP-LM2 were doped with [Str-(RL)_4_G-NH_2_] peptide 3 µL of 1 mM (P’, 3 mol.% from lipid quantity) for preparation of siRNA-AuNP-LM1-P’ and siRNA-AuNP-LM2-P’; and 10 µL of 1 mM (P’’, 10 mol.%) for preparation of siRNA-AuNP-LM2-P’’.

### 3.9. Characterization of Nanospecies (The Term Nanospecies Is Used for Designation of All Particles, without Emphasis at Their Structure)

On each step of nanoconstruction preparation, resulting nanospecies were characterized by electrophoretic mobility in agarose gel. To do this, samples containing 5 µL of nanospecies (0.5 pmol) and 0.5 µL glycerol/deionized water (1:1, *v/v*) were loaded into the wells of a 0.8% agarose in Tris-glycine buffer (250 mM Glycine, 25 mM Tris, pH 8.3). Electrophoresis was carried out for 30 min at 5 V/cm. Images were obtained using Epson Perfection 4990 Photo (Epson, Suva, Japan) scanner.

Hydrodynamic diameter and ζ potential of all nanospecies obtained were determined using Zetasizer Nano NS (Malvern Instruments, Malvern, UK).

### 3.10. Transmission Electron Microscopy

All nanospecies obtained in this study were examined by transmission electron microscopy (TEM). A drop of sample was adsorbed for 1 min on the copper grid covered with formvar film which was pre-stabilized using carbon evaporation. Then excess liquid was pulled back by filter paper, and a grid was placed for 10 s on a drop of 1% uranyl acetate or 2% phosphor-tungstic acid (pH 3.0), excess liquid was collected with filter paper.

Cell pellets for TEM examination were fixed in 4% paraformaldehyde, postfixed in 1% OsO_4_ and after routine dehydration were embedded in epon-araldit (SPI, West Chester, PA, USA) mixture. To obtain ultrathin sections, hard blocks were cut on Leica EM UC7 (Leica Microsystems, Wein, Austria) ultratome and routinely contrasted with uranyl acetate and led citrate.

All grids were examined in Jem 1400 TEM (Jeol, Tokyo, Japan) and digital images were collected with Veleta camera (EMSIS, Muenster, Gemany).

### 3.11. Cells

Human embryonic kidney (HEK) Phoenix cells with stable integration of green fluorescent protein gene GFP into the genome were obtained from FRC Institute of Cytology and Genetics SB RAS. The cells were maintained in Dulbecco’s modified Eagle’s medium (DMEM) (Life Technologies, Paisley, UK), supplemented with 10% heat-inactivated fetal bovine serum (FBS) (Life Technologies, Paisley, UK) and 1% PeniStrep (Life Technologies, Grand Island, NY, USA). The cells were cultured in 24-well tissue culture plates (TPP, Trasadingen, Switzerland) in a 5% CO_2_ humidified incubator at 37 °C.

### 3.12. Fluorescent Confocal Microscopy

The cells were seeded on a sterilized cover slips in 12-well plates at a concentration of 10^5^ cells per well. After reaching the 50% confluence of the monolayer, the culture media was replaced with full media containing the studied samples of the nanocomposites (siRNA-Au-LM1-P’ and siRNA-Au-LM2-P’). siRNA was obtained by annealing of oligoribonucleotides ORN2 and ORN3 (Table 2). Final concentration of siRNA during cell transfection procedure was 12.5 nM. The cells were incubated for 4 h at 37 °C in the CO_2_ incubator. Then, the media was replaced with fresh one and cells were incubated for 24 h at 37 °C in the CO_2_ incubator. After incubation, cells were washed three times with PBS and fixed with 4% paraformaldehyde in DMEM for 30 min, and washed twice with PBS. The cell-containing cover slips were mounted on the slides using ProLong Gold antifade reagent with DAPI (Life Technologies, Eugene, OR, USA). The slides were examined with confocal laser scanning microscope LSM 510 UV Meta (Carl Zeiss, Oberkochen, Germany). Three laser lines were used: 405 nm (to detect cell nuclei stained with DAPI), 488 nm (to detect GFP), and 633 nm (to detect Cy5.5 labeled siRNA).

### 3.13. Flow Cytometry

Efficiency of GFP gene silencing after transfection was quantified with flow cytometry via measuring of decrease of fluorescence intensity. Cells were grown in 24-well plate to achieve 50% confluence, and then cells were incubated for 4 h at 37 °C with: siRNA-Au; siRNA-Au-LM1; siRNA-Au-LM2; siRNA-Au-LM1-P’; siRNA-Au-LM2-P’; and siRNA-Au-LM2-P’’. Final concentration of siRNA was 12.5 nM. Cells transfected with Lipofectamine 3000 served as control for comparison. For this transfection, 6.25 pmol of siRNA in 50 µL of media without serum was mixed with 50 µL of media without serum containing 1.5 µL of Lipofectamine 3000. Obtained mixture was incubated 15 min and after added to cells in media without serum. Final concentration of siRNA was 12.5 nM. After the transfection, the cells were washed with PBS, and incubated in fresh full medium for 72 h. Then, the cells were washed with PBS, trypsinized and suspended in full medium. To detect the suppressing effect of siRNA on GFP synthesis, the cells were analyzed with a NovoCyte (ACEA Biosciences, San Diego, CA, USA) and NovoExpress software (ACEA Biosciences).

### 3.14. Statistical Methods

All binding probes were taken in triplicate, and error bars are shown on graphs only when calculated error value exceeds the marker size.

## Figures and Tables

**Table 1:** The concentration of the components for core preparation.

Sample	AuNPs (M)	siRNA (M)	Ratio siRNA/AuNPs
I	10^−7^	2 × 10^−5^	200
II	3 × 10^−9^	6 × 10^−7^	200
III	5 × 10^−10^	10^−7^	200

**Table 2:** Sequences of oligoribonucleotides.

Designation	Sequence
ORN1	5′-CAA-GCU-GAC-CCU-GAA-GUU-CTT-3′
ORN2	5′-GAA-CUU-CAG-GGU-CAG-CUU-GTT-3′
ORN3	5′-Cy5.5-CAA-GCU-GAC-CCU-GAA-GUU-CTT-3′
